# Muscle and tendon morphology alterations in children and adolescents with mild forms of spastic cerebral palsy

**DOI:** 10.1186/s12887-018-1129-4

**Published:** 2018-05-09

**Authors:** Annika Kruse, Christian Schranz, Markus Tilp, Martin Svehlik

**Affiliations:** 1 Institute of Sports Science, University of Graz, Mozartgasse 14, 8010 Graz, Austria; 2 Department of Paediatric Surgery, Medical University of Graz, Auenbruggerplatz 34, 8036 Graz, Austria

**Keywords:** Gastrocnemius medialis, Achilles tendon, Diplegic, Ultrasonography, Muscle fascicle

## Abstract

**Background:**

Early detection of changes at the muscular level before a contracture develops is important to gain knowledge about the development of deformities in individuals with spasticity. However, little information is available about muscle morphology in children with spastic diplegic cerebral palsy (CP) without contracture or equinus gait. Therefore, the aim of this study was to compare the gastrocnemius medialis (GM) and Achilles tendon architecture of children and adolescents with spastic CP without contracture or equinus gait to that of typically developing (TD) children.

**Methods:**

Two-dimensional ultrasonography was used to assess the morphological properties of the GM muscle and Achilles tendon in 10 children with spastic diplegic CP (Gross Motor Function Classification System level I–II) and 12 TD children (mean age 12.0 (2.8) and 11.3 (2.5) years, respectively). The children with CP were not restricted in the performance of daily tasks, and therefore had a high functional capacity. Mean muscle and tendon parameters were statistically compared (independent *t*-tests or Mann-Whitney *U*-tests).

**Results:**

When normalized to lower leg length, muscle-tendon unit length and GM muscle belly length were found to be significantly shorter (*p* < 0.05, effect size (ES) = 1.00 and 0.98, respectively) in the children with spastic CP. Furthermore, there was a tendency for increased Achilles tendon length when expressed as a percentage of muscle-tendon unit length (*p* = 0.08, ES = − 0.80) in the individuals with CP. This group also showed shorter muscle fascicles (3.4 cm vs. 4.4 cm, *p* < 0.01, ES = 1.12) and increased fascicle pennation angle (21.9° vs. 18.1°, *p* < 0.01, ES = − 1.36, respectively). However, muscle thickness and Achilles tendon cross-sectional area did not differ between groups. Resting ankle joint angle was significantly more plantar flexed (− 26.2° vs. − 20.8°, *p* < 0.05, ES = 1.06) in the children with CP.

**Conclusions:**

Morphological alterations of the plantar flexor muscle-tendon unit are also present in children and adolescents with mild forms of spastic CP. These alterations may contribute to functional deficits such as muscle weakness, and therefore have to be considered in the clinical decision-making process, as well as in the selection of therapeutic interventions.

## Background

Cerebral palsy (CP) is a well-recognized, common neuro-developmental disorder in children that describes a “group of permanent disorders of the development of movement and posture, causing activity limitation, that are attributed to non-progressive disturbances that occurred in the developing fetal or infant brain” [1]. CP causes secondary alteration of the musculoskeletal system, e.g., muscle weakness, restricted joint range of motion, and increased joint stiffness [2]; however, the basic mechanisms that lead to these functional deficits are still not clearly understood [3].

Due to their important relation to the functional capacity, recent studies have concentrated on the examination of both the function and the properties of the muscles and tendons in individuals with CP. These studies have reported critical changes within the muscles that cannot be explained by neural changes alone [3]. Consequently, improving the understanding of the alterations in spastic skeletal muscles is of particular importance.

The force-generation capacity of a muscle is, among other things, dependent on its morphological and architectural properties [4], as well as the morphology and the behavior of the corresponding tendon [5, 6]. Since ultrasound (US) imaging is non-invasive, inexpensive, accurately controlled, and accessible in most clinical environments [7], it is often used to examine muscle and tendon properties at a macroscopic level (e.g., fascicle lengths). Recently, US imaging has also been applied to determine the structural alterations of the commonly affected plantar flexors in children with CP [8–15], whereby the gastrocnemius medialis (GM) has often been the muscle of interest due to its functional significance and easy accessibility with US because of both its superficiality and short fascicles [16]. In agreement with studies that have used magnetic resonance imaging [17–19], studies performed with US imaging have reported consistent evidence of reduced muscle size, indicated by, among other things, reduced muscle volume [8, 11, 20, 21], thickness [9, 13], and belly length [8, 11, 15] in children with CP. Some of these alterations, e.g., the reduced muscle volume, have been found in very young children (2 to 5 years of age) [21]. However, studies concerning GM fascicle lengths have delivered inconsistent results, reporting shorter muscle fascicle lengths [9, 14, 22, 23] or no differences [8, 10] in individuals with CP. Investigations of the corresponding fascicle pennation angle have reported no difference between children with CP and typically developing (TD) children [8, 23], as well as between the paretic and non-paretic legs of hemiplegic individuals [9]. The reported inconsistencies might be related to differences in the examination methods of the studies, as well as the high inter-subject variability in children with CP. Furthermore, due to differences in etiology and motor impairment in individuals with CP, the inclusion of individuals with different sub-types (hemiparetic, diparetic) and/or with differences in gross motor function [9, 21, 23, 24] might have hampered the interpretation of the study results.

To date, the Achilles tendon morphological properties have not received much attention, and only a few studies are available that demonstrated increased Achilles tendon length and reduced tendon cross-sectional area in individuals with CP [23, 24]. The functional meaning of these alterations is not clearly understood, but they are considered an adaptation to the altered muscle architecture, to improve function [6]. Nevertheless, these changes may also contribute to muscle weakness [25].

In summary, alterations of the whole GM muscle-tendon unit architecture have been observed in individuals with spastic CP. Most of the previous studies have included severely impaired individuals (Gross Motor Function Classification System level ≥ III) [13], i.e., individuals with plantar flexion contractures [10, 15] and/or equinus gait [14, 24]. However, less information is available about children with spastic CP who do not suffer from contracture or equinus gait. In this context, Barber et al. [21] found reduced muscle volume and physiological cross-sectional area in very young children with CP (aged 2 to 5 years) without contracture, whereby the longer fascicle length and smaller pennation angle differed only at maximum plantar flexion. Furthermore, Wren et al. [24] reported alterations of both the GM muscle and the Achilles tendon (shorter belly length and longer tendon length, respectively) in individuals (~ 8.5 years) without contracture but with dynamic equinus gait. However, it is not well known if and how severely muscle-tendon morphology is altered in children and adolescents with mild forms of spastic CP.

Therefore, the purpose of this study was to examine the GM muscle and Achilles tendon architecture in children with mild spastic CP without muscle contracture or equinus gait, using US imaging, and to compare them to a group of age-matched TD peers. Based on previous results [21, 24], we hypothesized that muscle belly length would be reduced and the Achilles tendon lengthened in this group of children with CP who are not restricted in the performance of daily tasks. Furthermore, we assumed that we would find shorter muscle fascicles [24], as well as reduced muscle thickness [9], in the impaired children, but no differences in the pennation angles between groups.

## Methods

### Participants

Ten children with spastic diplegic CP (8–16 years; 5 girls and 5 boys) and 12 age-matched TD peers (7 girls and 5 boys) participated in the study. Six of the CP children were graded as GMFCS level I and four as level II. The children had no contracture of the plantar flexors (maximal ankle dorsiflexion ≥5° with knees extended) and did not present with equinus gait nor knee flexion deformities.

The exclusion criteria were other forms of CP than spastic CP, any previous surgery to the plantar flexors, and botulinum toxin application in the last six months. The children included in the present study who had received botulinum toxin A treatment received it nine months or more (range 9 months to 8 years) before the participation. Moreover, five out of the 10 children with spastic CP were botulinum toxin naïve. There were no differences in anthropometric data, e.g., body mass, body height, and lower limb length, between groups (Table 1). All participants were personally informed beforehand about the measurement procedure and parental written consent was obtained. The study was approved by the local ethics committee.

**Table 1:** Anthropometrics, ankle joint, and muscle-tendon parameters of children with cerebral palsy and typically developing peers

	CP (*n* = 10)	TD (*n* = 12)	Effect size
Age (years)	12.0 (2.8)	11.3 (2.5)	
Body mass (kg)	45.2 (19.1)	44.8 (15.5)	
Height (cm)	149.2 (21.2)	152.7 (16.5)	
Lower leg length (cm)	36.2 (6.0)	36.9 (5.3)	
Resting ankle joint angle (°)	−26.2 (5.7)*	−20.8 (4.5)	1.06
MTU length (normalized)^a^	1.06 (0.04)*	1.10 (0.04)	1.00
GM muscle belly length (normalized)^a^	0.53 (0.08)*	0.59 (0.04)	0.98
GM muscle belly length (% of MTU length)	50 (6)	54 (4)	0.80
Achilles tendon length (normalized)^a^	0.53 (0.06)	0.51 (0.05)	−0.37
Achilles tendon length (% of MTU length)	50 (6)	46 (4)	−0.80
Achilles tendon cross-sectional area (mm^2^)	44.5 (10.6)	52.3 (10.5)	0.74

Values are reported as mean (standard deviation). *CP* cerebral palsy; *TD* typically developing; *MTU* muscle-tendon unit; *GM* gastrocnemius medialis

^a^normalized to lower leg length

*significantly different from TD, *p* < .05

### Experimental protocol

All measurements were conducted by an experienced examiner. A standard US imaging system (MyLab60; Esaote S.p.A., Genova, Italy) was used for the image acquisition.

All measurements were made on the leg that demonstrated higher spasticity, as detected by clinical examination (Modified Ashworth scale) in the children with diplegic CP, and on the dominant leg of the TD children.

**Fig. 1:**
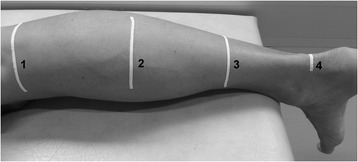
Measurement setup for the calculation of gastrocnemius medialis muscle and Achilles tendon morphological properties. Numbers 1 and 3 on the lower leg indicate the tape strips used to calculate the muscle belly and tendon length, number 2 shows the tape strip placed as a visual aid to determine 50% of the muscle belly length, and number 4 indicates the tape strip that was utilized as a marker to assess the Achilles tendon cross-sectional area

The lower limb length was first determined by the use of a measuring tape, defined as the distance from the malleolus lateralis to the epicondylus lateralis. For the further GM muscle-tendon unit length and architecture measurements, the children were asked to lie prone on an examination couch, with their feet hanging over the edge and knees fully extended (Fig. 1). US measurements were repeated three times, and the average values were used for the further analysis.

### Resting ankle joint angle determination

The resting ankle angle of each subject was measured by standard goniometry to further determine the Achilles tendon resting length. Therefore, one arm of the goniometer was kept parallel to the sole of the foot, and the other arm was kept in line with the line defined by the participant’s malleolus lateralis and epicondylus lateralis. The resting ankle position was defined as the relaxed position of the foot, with no external force applied by the examiner [8].

### Length measurements of the muscle-tendon unit

**Fig. 2:**
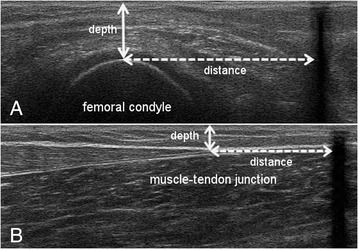
Ultrasound images used to assess the GM muscle belly and Achilles tendon lengths, based on the US-tape method of Barber et al. 2011. The dashed lines show the distance from the superficial aspect of the condyle (**a**) and the most distal aspect of the muscle-tendon junction (**b**) to the tape (black shadow), whereby the white arrows indicate the US depth

In order to determine the muscle-tendon unit, GM muscle belly, and Achilles tendon length at rest, 3 adhesive tape strips (Fig. 1) were placed on the skin close to predefined landmarks [26]: the most superficial aspect of the medial femoral condyle (Tape 1), the most prominent bulge of the GM (Tape 2), and the muscle-tendon junction of the GM and the Achilles tendon (Tape 3). A measuring tape was used to ascertain the distances between the calcaneus and each tape (distance calcaneus–tape). Subsequently, the US transducer was placed longitudinally over the respective landmarks, and images showing the landmark and the corresponding shadow of the tape in the US image (clearly visible due to its anechoic behavior, Fig. 2) were recorded. To determine the length measurements, a 10 cm linear-array probe (10 MHz; depth 74 mm; LA 923, Esaote, S.p.A) was used, and the focal depth was optimized to allow ease of identification of the structures [27].

Post-processing similar to the approach of Barber et al. [26] was performed by combining both the external tape measurements and the length information in the US images, determined by means of Tracker open-source software (version 4.91, http://physlets.org/tracker/). Firstly, the longitudinal distance between the respective landmark and the edge of the shadow of the tape in the US image (Fig. 2, distance) was added to the tape measurement. Secondly, the vertical distance between the upper boundary of the US image and the landmark (Fig. 2, depth) was used to calculate the linear distance from the landmark to the calcaneus with Pythagoras’ theorem. With regard to the landmarks (Fig. 2a, b, lateral femoral condyle and muscle-tendon junction), the described procedure allowed us to calculate the muscle-tendon unit length and Achilles tendon length, respectively.

GM muscle belly length was in turn calculated by subtracting the Achilles tendon length from the muscle-tendon unit length and, therefore, defined as the distance between the medial femoral epicondyle and the GM muscle-tendon junction. Image analysis was conducted for three images, resulting in 3 lengths for each parameter. The mean was defined as the muscle-tendon unit, GM belly, or Achilles tendon length, respectively. All lengths were normalized to lower leg length to account for any differences in body size [24]. Moreover, muscle belly and Achilles tendon length were further expressed as a percentage of muscle-tendon unit length [24].

### Measurement of GM muscle architecture and morphology

**Fig. 3:**
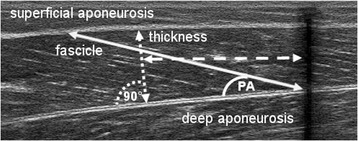
Ultrasound image of the GM muscle showing determination of muscle thickness (dotted line), fascicle length (solid line), and pennation angle (PA). Muscle thickness was measured at 50% of the muscle belly length (dashed line) determined from the echo of the applied tape (black shadow on the right side)

GM muscle morphological properties were assessed at rest. US images were captured to determine fascicle length, pennation angle, and muscle thickness, whereby the US probe was aligned perpendicular to the deep aponeurosis in order to find the true fascicle plane [28]. Fascicle length (Fig. 3) was defined as the linear distance between the insertion into the deep and superficial aponeurosis [8]. In each of the 3 US images, 3 fascicles that were identifiable throughout their whole length were chosen [14] and manually measured by following their paths in a straight line. As a result, the mean value of 9 fascicles was defined as the subject’s GM fascicle length. In contrast to previous studies [10], fascicles were visible over their whole length due to the size of the US probe (10 cm), which allowed direct length measurements without length calculation or use of an extrapolation method. Mean fascicle length was further normalized to the muscle-tendon unit and muscle belly length. The pennation angle (Fig. 3) was defined as the angle between the fascicle selected for length measurement and the deep aponeurosis [29], and was measured for each defined fascicle in each image. GM muscle thickness (Fig. 3) was measured at 50% of the muscle belly as the perpendicular distance between the deep and superficial aponeurosis [9]. In order to find the middle part of the GM muscle belly (50%), tape (Fig. 1) was used as a marker. The mean of 3 thickness values was used in the final analysis. The procedure applied to determine muscle fascicle length, pennation angle, and muscle thickness has been reported to be highly reliable [9].

### Measurement of the Achilles tendon cross-sectional area

To determine the Achilles tendon cross-sectional-area, transverse US images were recorded at the level of the medial malleolus. Therefore, the path from the tip of the malleolus to the tendon action line was quantified and a line was drawn (medio-lateral direction). Owing to the width of the footprint of the transducer (50 mm × 8 mm), a separate line was drawn 4 mm below the first and defined as the measurement position [30]. Similar to the muscle-tendon unit length measurements, an adhesive tape strip was fixed below the second line and used as the lower boundary to locate the selected measurement position (Fig. 1). To enhance the visibility of the tendon boundaries, a stand-off gel pad (SONOKIT soft 200 × 100 × 20 mm; SONOGEL, Bad Camberg, Germany) was placed between the skin surface and the US probe. In contrast to the length measurements, a shorter linear-array transducer (LA 523, Esaote, S.p.A; 10 MHz; 30 mm depth) was used.

**Fig. 4:**
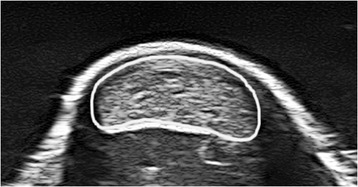
Transverse US image showing the manually outlined Achilles tendon cross-sectional area (white shape)

The transverse US images of the Achilles tendon cross-sectional area were analyzed using ImageJ software (version 1.48v; National Institutes of Health, USA). The cross-sectional area was manually outlined, excluding the dense connective tissue of the tendon (Fig. 4), and subsequently calculated by the software. Three images were digitized, and the mean value was defined as the cross-sectional area. The mean coefficient of variation for repeated measures was 1.9% [30].

### Statistical analysis

All the statistical analyses were performed using SPSS (version 22.0, SPSS Inc., Chicago, III). The level of significance was set to 0.05 for all the tests. Data are presented as means and standard deviations for each group, and independent t-tests were performed to test for differences between groups. Kolmogorov-Smirnov tests were used to test for normal distribution of the data. In the case of the data not being normally distributed, a non-parametric statistical test (Mann-Whitney U-test) was performed. Furthermore, effect sizes (ES, Cohen’s d) were calculated, where 0.2 characterizes a small effect, 0.5 a medium effect, and 0.8 a large effect [31].

## Results

The resting ankle joint was more plantar flexed (by ~ 5°) in the children with CP compared to the TD children (Table 1). When normalized to lower leg length, muscle-tendon unit length and GM muscle belly length were significantly shorter (*p* = 0.02, respectively) in the children with CP (Table 1). Furthermore, when expressed as a percentage of muscle-tendon unit length, there was a tendency for decreased muscle length and increased tendon length (*p* = 0.08, ES = 0.80 and − 0.80, respectively) in the CP group (Table 1).

GM muscle fascicles at rest were found to be significantly shorter in the children with CP compared to the TD group, in both absolute and normalized terms (ES = 1.12 and 1.42 respectively, Table 2). In addition, the CP group showed a significantly greater GM pennation angle (ES = − 1.36); however, GM muscle thickness did not differ between groups (ES = 0.00). Finally, no significant difference between the children with CP and the TD group could be found in the Achilles tendon cross-sectional area, although we did find a moderate effect (ES = 0.74, Table 1).

**Table 2:** Muscle morphology of the gastrocnemius medialis in children with cerebral palsy and typically developing children

	CP	TD	Effect size
Fascicle length (cm)	3.4 (1.0)*	4.4 (0.8)	1.12
Fascicle length (normalized to MTU)	0.09 (0.02)**	0.11 (0.02)	1.31
Fascicle length (normalized to muscle belly length)	0.19 (0.04)	0.20 (0.03)	0.29
Pennation angle (°)	21.9 (2.9)**	18.1 (2.7)	−1.36
Muscle thickness (cm)	1.3 (0.4)	1.3 (0.2)	0.00

Values are reported in mean (standard deviation). *CP* cerebral palsy; *TD* typically developing; *MTU* muscle-tendon unit

*significantly different from TD, *p* < .05

**significantly different from TD, *p* < .01

## Discussion

In this study, we used US imaging to investigate the GM muscle architecture and Achilles tendon properties in 10 children and adolescents with spastic diplegic CP without contracture or equinus gait and 12 TD peers. We found shorter muscle-tendon unit and GM muscle belly lengths in the individuals with CP, and a tendency for increased Achilles tendon length. Furthermore, shorter GM muscle fascicle lengths and increased GM fascicle pennation angle were also present in the CP group.

Muscle architecture crucially determines the amount of force, maximum shortening velocity, excursion of the muscle, and the force that is transmitted to the tendon [32]. Therefore, alterations of these properties may contribute to the reduced force output in individuals with CP. In the present study, we found reduced GM muscle belly lengths in children and adolescents with spastic CP without contracture or equinus gait, which is in good agreement with previous studies in individuals with diplegic and hemiplegic CP [8, 10, 15, 22, 24]. Reduced muscle lengths are commonly thought to be caused by a reduction in muscle fascicle length [33]; however, other explanations, such as a decrease in the mean muscle fiber diameter and, therefore, a shortening of the aponeurosis, have also been put forward [8, 10].

Although fascicle length did not differ between the groups when normalized to muscle belly length, absolute values and lengths normalized to overall MTU were smaller in children with CP therefore confirm previous results [9, 14, 22, 23]. This indicates that reduced GM muscle belly lengths may be a result of reduced fascicle lengths in children with CP. It is widely believed that the reduced fascicle lengths in CP are a result of a loss of serial sarcomeres [19, 34], and studies have consistently shown that sarcomeres in muscles of individuals with CP are longer [35] compared to sarcomeres in normally developed fascicles. In combination with an increased pennation angle, these alterations at the fascicular level will greatly contribute to the decreased force production in children with CP [24, 35].

The determined muscle fascicle pennation angles in the present study were found to be significantly increased in the CP group (TD: 18.1°; CP: 21.9°). This finding is in contrast to other studies [8, 10, 21] that have reported no differences between groups or slightly smaller pennation angles in children with CP. Nevertheless, our finding is in good agreement with the observation of Gao et al. [23], who concluded that, from a geometrical point of view, the pennation angle would increase under similar muscle thickness if a muscle fascicle shortens. Therefore, increases in fascicle pennation angle in children with CP may be an adaptation to a reduction in muscle fascicle length. Our findings support this assumption. Furthermore, recent studies have shown consistent evidence that GM muscle fascicle lengths vary systematically with ankle position [10, 22, 23]. Therefore, we suppose that this increase in fascicle pennation angle may also be related to the more plantar flexed resting ankle joint angle that we found in the CP group. Furthermore, there are several aspects that may explain the different pennation angle values between studies: different measurement locations within the muscle [9], high inter-subject variability for a given age and increases of this angle as a function of age [36], and gender-specific variability of the muscle dimensions [37]. Therefore, a comparison of pennation angle values between studies should be conducted with care.

We further assessed muscle thickness as an approximation of muscle size, due to its high correlation with muscle cross-sectional area [37, 38]. Against our hypothesis, we did not find any difference in muscle thickness between the children with CP and the TD group, which is also in contrast to other studies [9]. However, in contrast to the study of Mohagheghi et al. [9], the analyzed children with CP in the present study were less impaired with regard to motor function, which might have prevented the individuals from showing significant muscle atrophy.

With regard to the Achilles tendon, we found only a tendency for an elongated tendon in children with CP without contracture. Furthermore, we found a smaller (14%) Achilles tendon cross-sectional area in the CP group, but this result was also not statistically significant. Both findings are in line with previous studies that reported significantly longer Achilles tendons [22–24, 39] and significantly smaller tendon cross-sectional areas [23, 39] in more severely impaired children with spastic CP. It has been postulated that a longer and thinner Achilles tendon might be advantageous for energy storage [6] during movement. Therefore, the reported alterations of the tendon may be an adaptation to the altered muscle architecture (e.g. shortening of fascicles) to ensure or keep its function, whereby these structural changes might have a negative influence on joint control and lead to a reduction in muscle force [25]. Nevertheless, the children and adolescents with diplegic CP without contracture or equinus gait showed only a tendency for altered Achilles tendon properties. This finding might be explained by the relatively high activity levels of these individuals, which in turn might have caused a sufficient load to preserve normal tendon structure.

### Limitations

This study has several limitations that have to be considered when interpreting the results and/or comparing these results with other studies’ outcomes. Firstly, botulinum toxin A treatment within the 6 months before the examination was an exclusion criterion in this study. However, we are aware of some evidence in the literature that botulinum toxin A can introduce structural muscle changes that might last even longer than 6 months [40]. However, research on this topic, especially on humans, is still scarce and the results are inconsistent [40]. Moreover, 5 children out of the 10 did not receive botulinum toxin treatment before the study (Table 3). Therefore, we expect that the observed changes are related to the effects of CP, rather than its treatment with botulinum toxin.

**Table 3:** Previous botulinum toxin application (number, past months before study participation) in the medial gastrocnemius in the study participants with mild spastic CP

Subject	Number of injections	Months past from last injection
1	none	–
2	7	9
3	none	–
4	1	9
5	none	–
6	2	96
7	none	–
8	none	–
9	4	72
10	1	84

Another aspect that might raise some concerns is the matching of the groups. Despite the fact that we accounted for age, we did not match the groups for sex. Since Kawakami et al. [32] showed that muscle dimensions can vary with sex, we cannot say that this feature did not have an influence on the study outcomes, although the differences between groups were small (50% males vs. 42% females).

Furthermore, we have to note that conventional two-dimensional US imaging has several drawbacks (e.g., operator-dependency [7]), which have to be considered when interpreting the results. The limited dimensions of the transducer do not allow for accessing some important outcome measures such as muscle volume and/or the entire MTU [7]. Due to the fact that two-dimensional US imaging restricts the morphological measurements to one image plane and therefore involve error sources, it is necessary to strictly follow guidelines for the assessments of e.g. fascicle length [28] to increase the repeatability of the measurements. Despite the fact that the described limitations might be overcome by the use of new valid and reliable techniques (e.g. 3-dimensional freehand US imaging [41, 42]), their applicability for the clinical environment still has to be improved [7]. With regard to the procedures used in this study, we can report that all the assessments of muscle and tendon properties between the CP and TD groups were conducted in accordance, so that the possibly occurring errors were similar in both groups, and should therefore not affect the main outcomes of the study.

Additionally, we have to note that we performed the internal reliability analysis only for the assessment of the Achilles tendon cross-sectional area. Therefore, we cannot report on the reliability of the other outcome parameters, such as muscle fascicle length and pennation angle. However, previous studies have shown that the architectural properties of the GM muscle can be reliably assessed with the muscle in a relaxed state, and without formal training of the reviewer [43]. In this study, the measurements were performed by an investigator with two years of experience, and following the guidelines for US assessments [28]. Therefore, we have confidence in our results.

### Clinical implications

Muscle architecture is a crucial determinant of muscle force-generating capacity and its excursion [4], whereby muscle fascicle length is the primary determinant of muscle excursion, with shorter muscle fascicles having a reduced range through which they can develop force and power [4]. Furthermore, there is consistent evidence of reduced muscle size, as indicated by reduced muscle volume as well as muscle belly length, in the comparisons between spastic CP and typically developed muscles [e.g. 8, 11, 15, 17, 21]. These architectural features affect the muscle function and contribute to functional deficits such as plantar flexor weakness in individuals with CP. Therefore, to understand the changes in the architecture of spastic muscles is crucial information for planning treatment modalities such as stretching, orthotic management, or even surgery. Our study showed that muscle and tendon morphology is altered even in children with CP without muscle contracture and who are not restricted in their performance of daily tasks. These results may suggest that individuals with mild forms of CP are also at risk of developing contractures. Additionally, such individuals should also be included in interventional studies designed to counteract muscle weakness. Since alterations in GM muscle and Achilles tendon properties could be observed, we further suggest investigating the effects of treatments at both functional and musculo-tendinous levels.

## Conclusion

In conclusion, our findings show that architectural and morphological alterations of the GM muscle-tendon unit can also be found in children and adolescents with spastic diplegic CP without contracture or equinus gait. Therefore, we can confirm the previous results in younger individuals and children with CP without contracture but with equinus gait [21, 24]. Beyond the alterations reported for very young children without contracture [21], our findings may implicate deterioration of muscle alteration with growth, even in those children with mild CP.

As morphological properties are the main determinants of function, the observed alterations in muscle and tendon may contribute to functional deficits such as plantar flexor weakness. Reduced GM muscle fascicle lengths and increased fascicle pennation angles in individuals with CP may explain the decreased muscle belly length, as well as elongation of the Achilles tendon. The findings reported in the present study indicate the need to monitor the progression of muscle contracture.

## Abbreviations

CP: Cerebral palsy
ES: Effect size
GM: Gastrocnemius medialis
GMFCS: Gross Motor Function Classification System
MTU: Muscle-tendon unit
SPSS: Statistical Package for Social Sciences
TD: Typically developing
US: Ultrasound
