# Correction to: Muscle and tendon morphology alterations in children and adolescents with mild forms of spastic cerebral palsy

**DOI:** 10.1186/s12887-018-1251-3

**Published:** 2018-08-18

**Authors:** Annika Kruse, Christian Schranz, Markus Tilp, Martin Svehlik

**Affiliations:** 1 Institute of Sports Science, University of Graz, Mozartgasse 14, 8010 Graz, Austria; 2 Department of Paediatric Surgery, Medical University of Graz, Auenbruggerplatz 34, 8036 Graz, Austria

## Correction

Following publication of the original article [1], the author requested for an acknowledgement to retrospectively be added to the ‘Acknowledgements’ section of the article [1].

The addition is:

“The authors acknowledge the financial support by the University of Graz.”

Thank you.
